# Relationship between microcephaly and indicators of social inequality in the state of Paraíba, Brazil

**DOI:** 10.31744/einstein_journal/2023AO0043

**Published:** 2023-03-24

**Authors:** Anna Carolina D’ucarmo Bezerra, Manuela Leitão de Vasconcelos, Luiz Medeiros Araujo Lima, Daniele Andrade da Cunha, Giorvan Ânderson dos Santos Alves, Leandro Pernambuco

**Affiliations:** 1 Universidade Federal da Paraíba João Pessoa PB Brazil Universidade Federal da Paraíba, João Pessoa, PB, Brazil.; 2 Universidade Federal de Pernambuco Recife PE Brazil Universidade Federal de Pernambuco, Recife, PE, Brazil.

**Keywords:** Microcephaly, Zika virus, Zika virus infection, Health information systems, Epidemiology, Social indicators, Social determinants of health, Health inequities, Gender equity

## Abstract

**Objective:**

To analyze the relationship between microcephaly and social inequality indicators in the state of Paraíba during the biennium January 2015 and December 2016.

**Methods:**

Ecological study with data from newborn microcephaly records and municipal socioeconomic, environmental, and demographic indicators was conducted using two health information systems from the Brazilian Ministry of Health (SINASC and SINAN) and the Brazilian Institute of Geography and Statistics. A Poisson multiple regression model was applied with a significance level of 5%.

**Results:**

Among 223 municipalities in Paraíba, 74 registered new cases of microcephaly. The number of Zika virus cases, number of inhabitants, number of households without adequate water supply, and household income were predictor variables of the number of new cases of microcephaly in Paraíba.

**Conclusion:**

Microcephaly is associated with indicators of social inequality in Paraíba. Zika virus cases, water supply, and family income are the indicators that best explain the increase in microcephaly cases. Therefore, these variables must be monitored by health professionals and authorities.

## Highlights

• Seventy-four (33.18%) municipalities in the state of Paraíba registered live births with microcephaly.

• The highest proportion of cases (23.03%) was concentrated in the capital, João Pessoa.

• Number of inhabitants, number of cases of Zika virus, water supply, and average household income were associated with a higher proportion of new cases.

**Figure:**
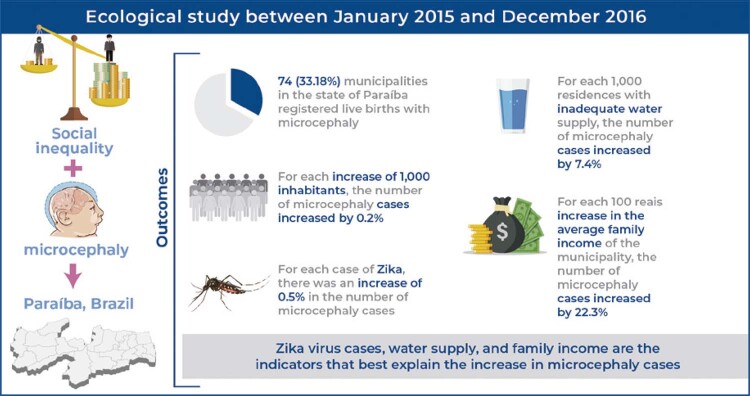


## INTRODUCTION

The year 2015 was marked by an unexpected increase in the number of microcephaly notifications in Brazil, particularly in the northeast region.^( 1 )^ During this period, it was necessary for the Ministry of Health to declare a public health emergency of national importance to investigate the identified cases.^( 2 , 3 )^ After epidemiological and laboratory studies, it was possible to verify the presence of Zika virus RNA in the amniotic fluid of newborns with microcephaly, constituting what started to be referred to as Congenital Syndrome of Zika Virus (CSZV).^( 4 , 5 )^

Microcephaly is defined as a congenital malformation characterized by decreased head circumference in relation to gestational age.^( 3 )^ The diagnosis was made from specific examinations and measurements of the cephalic perimeter using a nonelastic tape measure.^( 6 )^ Severe microcephaly is characterized by an alteration of two or three standard deviations below the expected average for gestational age and sex.^( 6 )^

Children with microcephaly may present with disorders in overall development, motor function, hearing impairment, language and speech delay, and feeding difficulties.^( 7 )^ Therefore, these children depend on access to rehabilitation services and need long-term therapeutic monitoring with special attention paid to social inequalities, since the patients’ families are often on low income and education.^( 8 )^

Social inequality can be understood as the process of unequal distribution of access to urban resources and services among groups that occupy different spaces.^( 9 )^ Previous research indicates a relationship between social determinants and the increase in the incidence of the Zika virus and its consequences, such as the CSZV,^( 10 )^ especially because the vector mosquitoes develop in standing water and are highly adapted to the environment of cities.^( 9 )^

It is known that in conditions of social and economic vulnerability, poor income distribution, and precarious housing, it is more difficult to access health services, resulting in a consequent increase in exposure to the diseases.^( 10 )^ Understanding these conditions is essential for proposing strategies to address health challenges. In Paraíba, the relationship between microcephaly case notification and municipal socioeconomic and environmental variables during the Zika virus epidemic is poorly understood. Investigating this relationship is essential to identifying the most vulnerable municipalities that need more attention regarding assertive and effective measures for the control and monitoring of the disease.

## OBJECTIVE

To analyze the relationship between microcephaly and indicators of social inequality in the state of Paraíba in the biennium January 2015 and December 2016.

## METHODS

This was an ecological study conducted with data from the state of Paraíba and its 223 municipalities, spread over an area of 56,584.6 km^2^ , and located in the northeast region of Brazil.^( 11 )^ The state had an estimated population of 4,039,277 inhabitants in 2020 and a Human Development Index of 0,658 in the year 2010.^( 11 )^

Data for this study were collected from the Department of Informatics of the Unified Health System (DATASUS - *Departamento de Informática do Sistema Único de Saúde* ) and organized using the public domain tabulator of the Ministry of Health (TABNET).^( 12 )^ In DATASUS, data were extracted from the *Sistema de Informação de Nascidos Vivos* (SINASC) and from the *Sistema de Informação de Agravos de Notificação* (SINAN). Other data were obtained using the Automatic Retrieval System of the Brazilian Institute of Geography and Statistics (SIDRA-IBGE; *Sistema de Recuperação Automática - Instituto Brasileiro de Geografia e Estatística* ). The dependent variable of the study was the number of new cases of microcephaly with no restriction due to etiology in each municipality of the Paraíba State in the biennium January 2015 and December 2016. The independent variables were the number of new Zika virus cases, the estimated number of inhabitants, the number of households with inadequate garbage collection, the number of households with inadequate water supply, the number of households with inadequate sanitation facilities, illiteracy rate, household income, Gini index, income ratio, and unemployment rate.

The dependent variable of the study was collected in SINASC considering the number of diagnoses of microcephaly ( *Classificação Internacional de Doenças* - CID-10 Q02) and according to the municipality of residence, in the years 2015 and 2016. Data from both years were added to compose a single variable for microcephaly cases in the biennium of interest.

In SINAN, we obtained the number of confirmed cases of Zika virus per municipality reported in 2016, considering the year of the first symptom (2015 or 2016). Confirmed cases of Zika virus in SINAN are based on laboratory criteria (viral isolation; reverse transcription polymerase chain reaction [RT-PCR] or indirect immunoglobulin M [IgM] serology) or clinical-epidemiological criteria.

In the tab “Health Information (TABNET)” of DATASUS, the options “Demographic and Socioeconomic” and “Labor and income - 1991, 2000 and 2010 census” were selected. The following variables were collected: average household income *per capita* , Gini index of household income *per capita* , income ratio, and unemployment rate 16a and +. The “Sanitation - 1991, 2000, and 2010 Census” option collected data on the number of households without garbage collection, the number of households with sanitation facilities without a general sewage network or septic tank, and the number of households with water supply without a network, well, or spring. Data from the 2010 Census were included as they were the closest to the studied biennium. In the SIDRA-IBGE, the variables collected were the illiteracy rate per municipality and the estimated number of inhabitants per municipality in the state of Paraíba in 2015.

In the descriptive analysis, measures of position and dispersion are presented. For the inferential analysis, a multivariate Poisson logistic regression model was used. All variables were included in the model and removed individually if the p-value was above 0.2. The R software was used with a significance level of 5%.

The submission of this study to the Ethics Committee for Research with Human Beings was dismissed because the research used a public domain database, without identification of individuals or exposure to any individual or collective physical or moral damage, as recommended by Resolution 466/2012, from the Brazilian National Council of Health.

## RESULTS

The descriptive analysis showed that of the 223 municipalities in the state of Paraíba, 74 (33.18%) registered 191 live births with microcephaly, with 120 (62.80%) in 2015 and 71 (37.20%) in 2016. The number of cases per municipality ranged from one to 44; however, most of the 74 municipalities (n=41; 55.41%) recorded only one notification ( Table 1 ). The highest proportion of cases was concentrated in the capital, João Pessoa, with 44 of the 191 notifications (23.03%), followed by Campina Grande with 11 (5.75%) and Cabedelo with seven (3.66%) cases.

**Table 1:** Distribution of microcephaly cases in the municipalities of Paraíba

Number of microcephaly cases	Number and proportion of municipalities n (%)
1	41 (55.41)
2	16 (21.62)
3	5 (6.76)
4	5 (6.76)
5	3 (4.05)
6	1 (1.35)
7	1 (1.35)
11	1 (1.35)
44	1 (1.35)

Source: Brasil. Ministério da Saúde. Banco de dados do Sistema Único de Saúde: Brasília (DF): Ministério da Saúde; 2021 [citado 2021 Jun 2]. Disponível em: https://datasus.saude.gov.br/^(12)^

Table 2 presents the measures of position and dispersion of the study variables in the 74 municipalities of Paraíba that presented with at least one case of microcephaly in the biennium 2015-2016. In the table, it is possible to observe high amplitudes in all variables, which illustrates the variability of these characteristics in the municipalities of Paraíba, Brazil.

**Table 2:** Measures of position and dispersion of study variables

Variable	Minimum	Maximum	Amplitude	Median	Q25-Q75	Mean	Standard deviation
Cases of microcephaly	1	44	43	1.00	1.00-2.00	2.58	5.17
Confirmed cases of Zika virus	0	600	600	2.00	0.00-13.00	40.76	114.10
Estimated number of inhabitants	2,475	791,438	788,963	14,641	6,546-23,326	36,123	102,347
Number of households with inadequate garbage collection	730	20,977	20,247	4,684	2,329-7,153	5,268	3,589
Number of households with inadequate water supply	485	14,222	13,737	3,809	1,853-6,006	4,355	2,967
Number of households with inadequate sanitary facilities	1,282	209,579	208,297	8,879	4,546-14,627	14,877	26,942
Illiteracy rate	7.70	40.70	33	28.75	23.75-33.25	28.06	7.30
Unemployment rate	1.64	19.79	18.15	7.17	5.20-9.22	7.48	3.22
Average family income (in Brazilian *reais* )	179	959	780	262.50	232.75-317.50	302.12	132.65
Income ratio	12.73	78.16	65.43	23.10	19.08-28.33	25.27	9.72
Gini Index	0.44	0.70	0.26	0.51	0.51-0.54	0.52	0.04

Source: Brasil. Ministério da Saúde. Banco de dados do Sistema Único de Saúde: Brasília (DF): Ministério da Saúde; 2021 [citado 2021 Jun 2]. Disponível em: https://datasus.saude.gov.br/^(12)^

The final regression model indicated that the socioeconomic and environmental indicators that best explained the number of new cases of microcephaly in the state of Paraíba in the biennium 2015-2016 were the estimated number of inhabitants of the municipality, the number of cases of Zika virus, water supply, and average household income ( Table 3 ).

**Table 3:** Final Poisson regression model with socioeconomic and environmental indicators

Variables	Estimated parameter	Standard error	p value	Odds ratio
Intercept	0.4145	0.2590	0.1095	0.6606
Population	0.0023	0.0005	<0.0001	1.0023
Zika virus cases in 2016	0.0053	0.0018	0.0043	1.0053
Water supply	0.0176	0.0241	0.0029	1.0742
Family income	0.0716	0.0613	0.0010	1.2231

Significant p value if <0.05.

For the purpose of interpreting the variables, for each increase of 1,000 inhabitants, the number of microcephaly cases increased by 0.2%; for each case of Zika, there was an increase of 0.5% in the number of microcephaly cases; for each 1,000 residences with inadequate water supply, the number of microcephaly cases increased by 7.4%; for each 100 *reais* increase in the average family income of the municipality, the number of microcephaly cases increased by 22.3%.

## DISCUSSION

Microcephaly is a congenital malformation with high individual and family impact, which gained notoriety between 2015 and 2016 in Brazil due to the births of children with CSZV.^( 13 , 14 )^ This study investigated microcephaly in live births from an ecological perspective, and found that strategies for health promotion, basic sanitation, and more balanced income distribution should be developed to reduce and monitor microcephaly cases in the state of Paraíba.

The city of João Pessoa, the capital of the state, had the highest estimated number of inhabitants per city and the highest number of reported cases of microcephaly during the study period. Although many areas of João Pessoa deal with precarious sanitary conditions, this result was expected because it is generally observed that in capital cities, the number of inhabitants is high, and is associated with a larger and better network of health coverage and active epidemiological surveillance, especially in terms of detection, notification, and monitoring of cases.^( 15 )^ Previous study identified that in Paraíba, as well as in the states of Rio Grande do Norte and Bahia, the cases of microcephaly and Zika virus were highly concentrated in the capital cities of the states, which are municipalities better served by health units and have greater coverage of diagnostic resources and health professionals.^( 16 )^

The relationship between microcephaly and the number of Zika virus cases can be understood by analysing the beginning of notifications of these cases in the northeast region in 2015. The Zika virus dissemination and infection of pregnant women in the Northeast region can be explained by socio-environmental factors, since the vector of the virus, *Aedes aegypti,* proliferates in places with standing water and inadequate garbage collection.^( 17 )^

It is known that inappropriate disposal of garbage can directly impact the health of an entire population, because proper garbage care prevents the proliferation of diseases, whether in urban or rural areas.^( 18 )^ Although data on the absence or inadequate collection of garbage was not included in the final regression model of this study, it is understood that the inadequate disposal of garbage can contribute to the accumulation of water, which favors mosquito breeding.^( 19 )^

According to this study, inadequate water supply was one of the predictive factors for the number of live births with microcephaly in the state of Paraíba. In addition to preventing diseases and reducing negative health impacts, adequate water supply conditions are associated with a longer life expectancy and reduced mortality.^( 20 )^ A study carried out in Campina Grande, the second most populous municipality in the state of Paraíba, identified that the city has flaws in the water distribution system, as confirmed in this study; our data showed that this municipality had one of the least adequate water supply systems.^( 21 )^ Poor water supply conditions are also related to the socioeconomic conditions of the population, since inadequate housing standards and low incomes have been associated to poor sanitation and water distribution conditions, in addition to gaps in the implementation of adaptation measures in households.^( 21 )^

Regarding socioeconomic aspects, the municipalities of Campina Grande, João Pessoa, and Cabedelo had the highest average household incomes, indicating that these cities have more resources for the diagnosis and notification of microcephaly cases. A study conducted in the state of Rio Grande do Norte found a relationship between average household income and the average incidence rate of the Zika virus in 2015 and 2016, which was attributed to better access to health services found in capital cities and metropolitan regions.^( 10 )^ However, it should be recognized that this variable has limitations as an explanatory variable for the outcome. We highlight that the study was carried out considering the comparison between municipalities and that the predictive value of average household income may be limited in identifying social inequalities within the municipality itself.

This study had some limitations. The absence of a georeferenced analysis compromises the acquisition of information at more specific geographic levels, which could generate different results. The statistical model in this study used municipalities as the unit of analysis; therefore, the results cannot be extrapolated to the individual level. Another limitation is secondary data sources, which may suffer from inconsistencies in data collection, registration, availability, and continuity. Some variables included data collected from the most current census, which did not always correspond to the same period of interest of this study.

## CONCLUSION

This study found a relationship between the indicators of social inequality and microcephaly in the state of Paraíba in the biennium 2015-2016. The distribution of socioeconomic and environmental indicators was unequal among the municipalities of Paraíba, with notifications of microcephaly in live births during the study period. Higher Zika virus rates, a higher number of inhabitants, more households without adequate water supply, and a higher average income were predictors of the increase in microcephaly cases in the state. Public policies should promote greater equity among the municipalities of Paraíba regarding socioeconomic and environmental variables associated with microcephaly, including monitoring and prevention of Zika virus cases, education of the population, and better water supply conditions.
